# Low-cost formation of bulk and localized polymer-derived carbon nanodomains from polydimethylsiloxane

**DOI:** 10.3762/bjnano.6.76

**Published:** 2015-03-16

**Authors:** Juan Carlos Castro Alcántara, Mariana Cerda Zorrilla, Lucia Cabriales, Luis Manuel León Rossano, Mathieu Hautefeuille

**Affiliations:** 1 Departamento de Física Facultad de Ciencias, Universidad Nacional Autónoma de México, Avenida Universidad 3000, Circuito Exterior S/N, Delegación Coyoacán, C.P. 04510 Ciudad Universitaria, D.F. México, México

**Keywords:** carbon nanodomains, nanodots, polydimethylsiloxane, polymer-derived ceramics, Raman spectroscopy

## Abstract

We present two simple alternative methods to form polymer-derived carbon nanodomains in a controlled fashion and at low cost, using custom-made chemical vapour deposition and selective laser ablation with a commercial CD-DVD platform. Both processes presented shiny and dark residual materials after the polymer combustion and according to micro-Raman spectroscopy of the domains, graphitic nanocrystals and carbon nanotubes have successfully been produced by the combustion of polydimethylsiloxane layers. The fabrication processes and characterization of the byproduct materials are reported. We demonstrate that CVD led to bulk production of graphitic nanocrystals and single-walled carbon nanotubes while direct laser ablation may be employed for the formation of localized fluorescent nanodots. In the latter case, graphitic nanodomains and multi-wall carbon nanotubes are left inside microchannels and preliminary results seem to indicate that laser ablation could offer a tuning control of the nature and optical properties of the nanodomains that are left inside micropatterns with on-demand geometries. These low-cost methods look particularly promising for the formation of carbon nanoresidues with controlled properties and in applications where high integration is desired.

## Introduction

Silicon-based polymer-derived ceramics (PDC) are of increasing interest thanks to the relatively controlled production and recent utilization of their unique functional properties. Their great resistance and stability to high temperatures, chemical durability or semiconducting behaviour are indeed permitting new applications as catalysis supports, highly resistant materials for aerospace and automotive industry, microelectronics, optics and even biotechnology [1]. Recent efforts have been made to properly control and characterize the production of various forms of PDC pyrolysing different preceramic polymers. Coatings, fibres and orderly porous components may then be obtained according to the desired application and nanodomains such as carbon nanotubes and nanowires, silicon carbide (SiC) and SiC/SiO2 nanofibres have also been recently produced [2–3]. Typically, the use of special fillers and high temperatures of 1000 °C or greater are critical for the kinetics, morphology, composition and organization of the resulting residual nanostructures. Moreover, the complex nature of preceramic silicon-based polymers may also represent a limitation to the formation of PDC nanodomains. It has been reported that carbon nanostructures may be obtained through the chemical vapour deposition (CVD) technique by using alcohols as reagent for carbon sources. For instance, aliphatic alcohols or mixtures of ethanol and methanol with other substances such as ferrocenes may also be used, depending on the type of the desired carbon nanodomains [4–5]. Laser pyrolysis may also be used to produce PDC in a rapid, local and selective fashion, although it is less common than CVD due its non-continuous work regime limiting the process [1]. Recently, a high power ultraviolet laser has been employed to directly induce the localized formation of nanocrystalline silicon residues in a low-cost polymer matrix with many interesting properties in optics and electronics micro-integration: polydimethylsiloxane (PDMS) [6].

In this work, we report the formation of bulk or localized carbon nanodomains obtained from PDMS, a silicon-based polymer by using two different methods enabled by low-cost custom-made platforms: chemical vapour deposition at 900 °C and selective laser ablation (SLA). In both procedures, it has been found that the combustion of PDMS successfully formed residual nanomaterials of different compositions and ordering depending on the formation conditions. Ease of use at atmospheric pressure and low cost are characteristics of the two alternative techniques employed here for PDC nanodomains production. In CVD, the evaporation of the reagent over the PDMS at high temperatures allowed for the rearrangement of carbon structures in PDMS to produce two layers composed of two different carbon structures. In the case of SLA, a continuous-wave infrared laser beam was focused on the surface of PDMS membranes coated with a thin layer of absorbing carbon nanopowder. This generates superficial laser-induced incandescence and forms highly localized carbon nanodomains inside the microscopic volume of etched material [7]. In this particular case, the PDC organization was highly dependent on lasing conditions and the process proved to be useful to produce localized fluorescent nanodomains in a PDMS matrix with a direct, controlled, rapid-prototyping method, similar to other SLA methods [8].

## Results and Discussion

### Chemical vapour deposition

**Figure 1:**
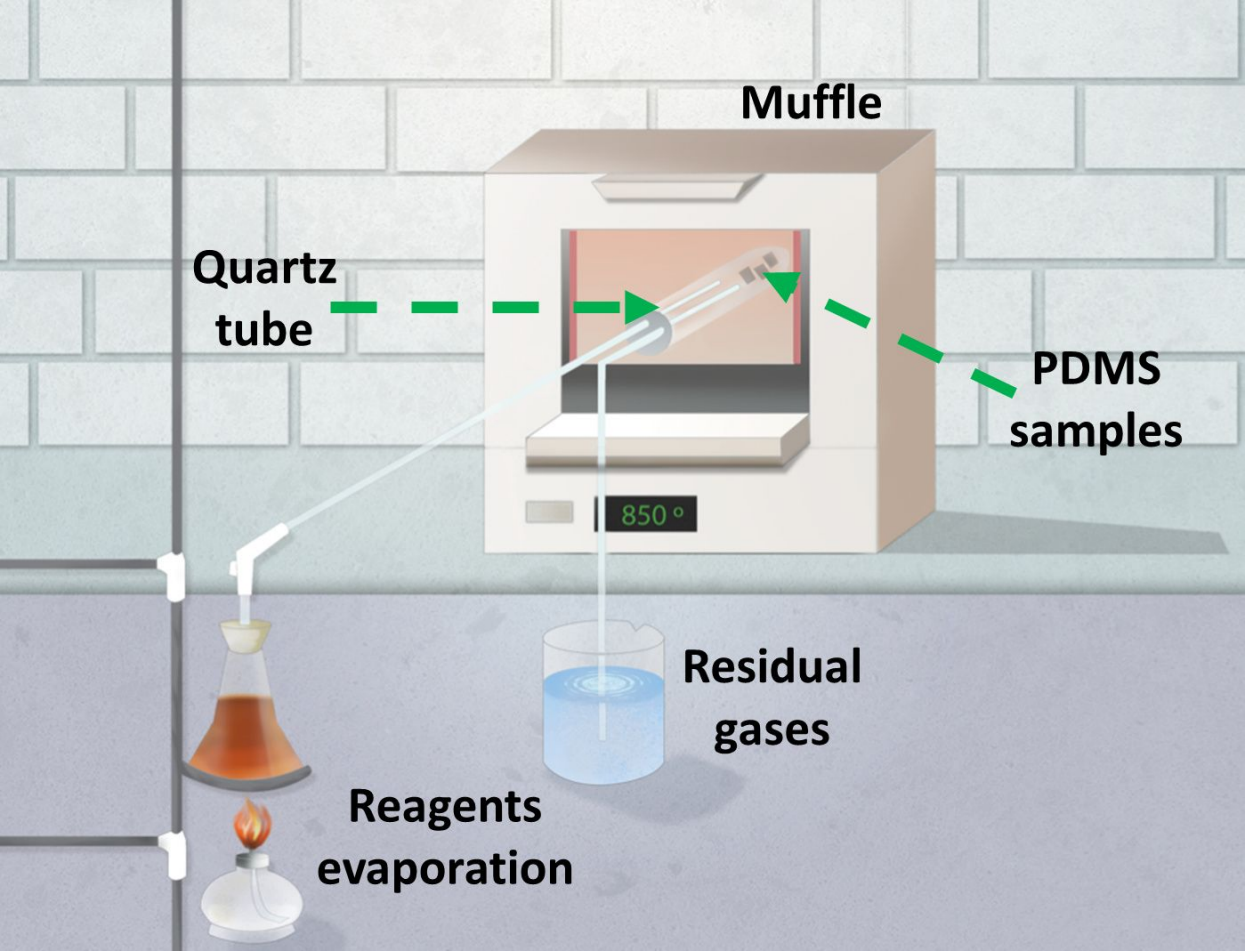
Diagram of the custom-made atmospheric pressure CVD reactor.

When a commercial CVD reactor is lacking, the low-cost alternative constructed here using conventional laboratory materials showed to be capable of vapour deposition at atmospheric pressure. The setup consists of three essential parts: reagents evaporation stage, deposition chamber and residual gases expulsion stage, as shown in Figure 1. The reagents evaporation is based on a heating system and a glass recipient connected to the deposition chamber through a valve. The deposition chamber consists of a quartz tube located inside a muffle (Lindberg 51894), sealed by a two-hole rubber stopper that allows for gas transport both inwards and outwards. Residual gases expulsion is verified by their bubbling in a water container placed outside the chamber.

The reagent used in this work for nanodomains production was a solution of ethanol (laboratory reagent purchased from Sigma-Aldrich, Part No. 16368) in water at 43% (v/v). The 3 mm thick PDMS layers on which it was evaporated were prepared from a Sylgard 184 kit (purchased from Dow Corning) mixing the pre-polymer with its curing agent in a 10:1 weight proportion following the conventional procedure. The gel was then deposited on clean glass substrates and cured at 90 °C for 2 h in a convection oven before they were detached and stored in hermetic containers. Vapour deposition of the reagent was performed at 900 °C over one hour and temperature was measured with a type-K thermocouple placed inside the deposition chamber. A post-treatment consisting in heating the sample at the same temperature for 30 min has been implemented to improve deposition and ordering of the structures [9]. After this procedure, the quartz tube was removed to let the chamber cool down to ambient temperature and the samples were carefully extracted with tweezers due to their brittle nature.

**Figure 2:**
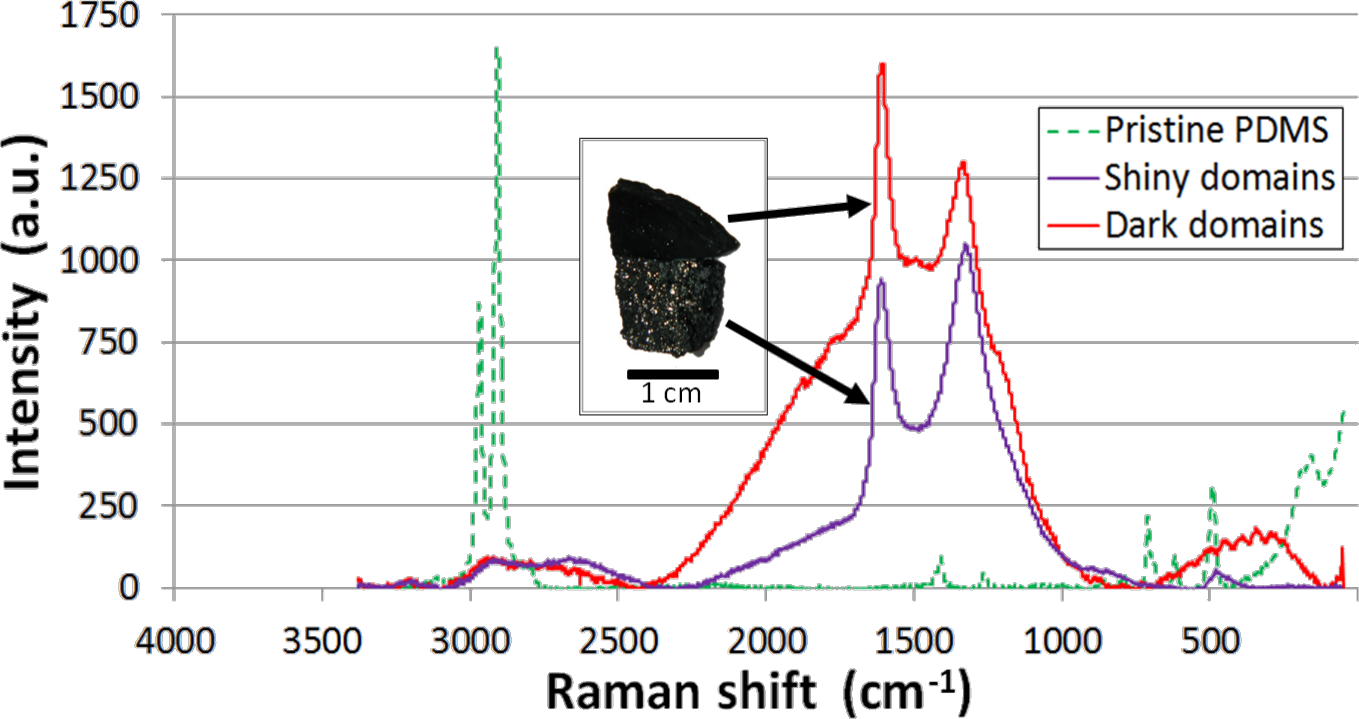
Comparison of Raman spectra: pristine PDMS, carbon nanotubes (shiny domains) and graphite nanocrystals (dark domains).

Two very distinct types of residual layers were always observed after the CVD procedure: dark and shiny layers have been obtained in a highly repeatable manner under the previously described particular conditions (Figure 2). Different geometries and shapes have been used and it did not affect the results. The totality of the pristine PDMS layers was then transformed into these two layers, proving that this method is appropriate for bulk production of these residues. The Raman spectra of these optically different materials, obtained with a Raman microscope with a 532 nm laser of (Thermo Scientific DXR), showed that the layers obtained with the CVD technique are very different from that of a pristine PDMS substrate, as seen in Figure 2. In both cases, the D (ca. 1350 cm^−1^) and G (ca. 1598 cm^−1^) bands, characteristics of carbon materials, are clearly present. However, the G′ band (ca. 2700 cm^−1^) is visible in the dark regions while the 2D band (ca. 2700 cm^−1^) and the G + D band (ca. 2935 cm^−1^) are characteristics of the shiny areas [10]. The difference in visual appearance may then be also justified by a shift between these bands intensities, identifying distinct structures at nanoscale for each of the residual materials. Indeed, according to the literature [11–13], the greater intensity of the D band with respect to the G band and the presence of the RBM band (ca. 270 cm^−1^) demonstrate that graphitic nanocrystals (GNC) are composing the shiny layers, whereas dark parts consist mainly of single-walled carbon nanotubes (SWCNT) [14].

### Selective laser ablation

Selective and localized laser pyrolysis has also been tested to study the formation of nanodomains from PDMS in a controlled fashion, using a low-cost technique that allows laser micropattern formation in PDMS [15]. The setup used to locally etch polymer layers at the surface is based on a commercial CD-DVD optical pickup unit (OPU) mounted on a controllable platform [15]. It also allows for the precise control of laser power density and dwell time to control the formation of nanodomains. In spite of PDMS transparency at OPU available wavelengths, laser ablation has been achieved by coating the cured PDMS surface with absorbing carbon nanopowder clusters (Sigma-Aldrich, Part No. 633100) and focusing the infrared beam (785 nm) onto it above a certain power density threshold to generate localized laser-induced incandescence. The elevated heat generated by this phenomenon at the surface of PDMS is sufficient to generate a microplasma that promotes the combustion of the silicon-based elastomer and removes a microscopic volume of the polymer. Under certain controllable conditions, the combustion produces fumes and leaves black, fluorescent residual materials inside the resulting etched patterns [15]. Finally, the etched volumes are cleaned by using distilled water or common organic solvents that do not dissolve nor swell PDMS and remove additional nanopowders.

The SLA process produced nanocarbon residues inside micron-scale laser-etched PDMS channels with on-demand geometry. Dark and shiny domains were also observed by using polarized-light microscopy during which it was evidenced that the laser formation of the nanodomains is extremely localized. This has been clearly identified by the presence of fluorescent compounds inside the channels. The composition of the combustion residues has been characterized first with a NORAN energy dispersive X-ray spectroscopy (EDX) system working at 20 keV in a scanning electron microscope (Jeol JSM-5600LV). The EDX analysis showed that residues are mainly silicon and oxygen, in similar weight proportions, and that carbon is only present in regions etched at the greatest laser intensities. In this case, 47.5% oxygen, 29.3% silicon and 23.2% carbon are found inside the channels demonstrating that greater temperatures reached during incandescence produce carbon nanodomains locally.

**Figure 3:**
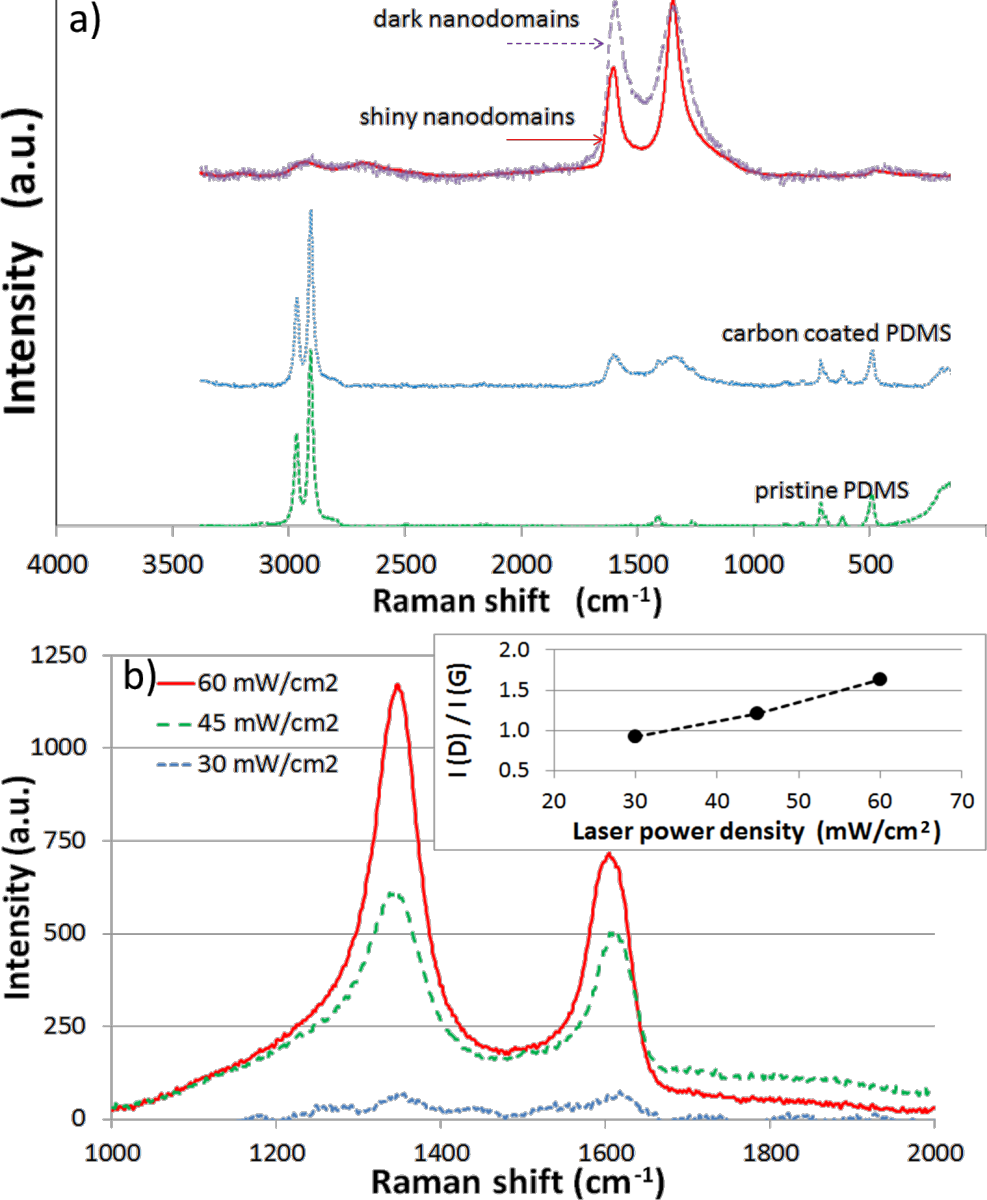
Raman characterization spectra of pristine PDMS, coated PDMS and residual nanodomains of shiny and dark domains in etched channels after cleaning (a). Influence of laser power density on G and D bands intensities (b).

The composition of the residues was then studied with Raman spectroscopy (Figure 3). This characterization showed residual materials structures inside microchannels similar to what was found after bulk PDC formation by using the CVD method. Namely, graphitic nanocrystals were found in shiny areas (demonstrated by the presence of D, G and 2D bands) while multi-wall carbon nanotubes (MWCNT) were found in dark regions (with the apparition of D, G and G′ bands, but without RBM band) [16]. It is interesting to remark that the laser-etched residual materials are slightly different from those obtained from CVD. No silicon oxides have been observed either, although it has been reported that they are typical residues of PDMS combustion [17]. This absence could be explained by the thermal conditions for PDMS ablation, since factors such as transient and final temperature during laser exposure greatly influence the nature of the byproducts as described in [17–19]. It has also been observed that the organization of carbon nanodomains is greatly affected by laser power density, thus by the temperature caused by the nanocarbon absorption of laser light (Figure 3b). This seems to indicate that the thermal conditions also play an important role in the nature of this type of byproducts [19]. Although the temperature reached on the PDMS surface has not been measured, the control of lasing conditions offered high process repeatability to obtain carbon nanodomains in a controlled fashion and with greater order than those obtained with a high-power excimer laser [6]. This shows that further work is needed in monitoring and controlling the thermal parameters of our SLA process. Finally, fluorescence has been observed inside the etched channels and it has been shown that its intensity is very much dependent on the laser power density used to etch the patterns, as described earlier in [15]. As fluorescence is visible after cleaning the samples, some nanoresidues are clearly embedded inside the polymer matrix, similar to what is reported in [20]. The nanoresidues also show good photothermal stability as the intensity of the fluorescence emission did not present quenching after hours of irradiation under a fluorescence microscope or when characterized with a 4 W UV lamp and characterized with a photomultiplier tube, even months after their formation. Unfortunately, the dependence of the fluorescence wavelengths and surface chemistry of the nanodomains with laser conditions as well as the thermal conditions influence on nanodomains formation have not been studied and will require future work to address these important parameters and to verify if our methods can be a low-cost solution for the tunable formation of carbon nanodots.
